# Inspecting EFL teachers' academic literacy development in multilingual contexts: A global vision

**DOI:** 10.1016/j.heliyon.2022.e12143

**Published:** 2022-12-07

**Authors:** Jia Fu, Yongliang Wang

**Affiliations:** a School of Medicine, Henan University, Kaifeng, China; b School of College English Teaching and Research, Henan University, Kaifeng, China

**Keywords:** Academic literacy, Academic literacy development, EFL teacher, Multilingualism, Multiculturalism, Second/foreign language education

## Abstract

Academic literacy, as a representation of required skills in the academic community, has gained increasing prominence and attention over the past decade. It has been the focal point of many studies in different contexts drawing on different perspectives. However, the way academic literacy development is researched and practiced in multilingual and multicultural settings is limitedly clarified and dealt with in second/foreign language contexts. Moreover, the role of different developmental trends of this research strand on EFL teachers in multilingual milieus has been overlooked, to date. To shed more light on the construct of academic literacy development in multilingual and EFL contexts, the present review article takes a global vision and explicates the past, present, and future trends in researching and practicing this significant construct. In so doing, it defines the concepts and presents the dimensions of academic literacy and academic literacy development. Then the role of academic literacy in multilingualism has been elaborated on using empirical evidence. Finally, the study provides and explains some implications for EFL teachers, teacher educators, curriculum designers, and scholars, who can update their information about academic literacy development in multilingual contexts with particular nuances.

## 1 Introduction

With the fast pace of English language teaching and learning in the globalized era, the importance of academic literacy skills has exponentially enhanced in different contexts, especially second/foreign language education (Canagarajah, 2020; Muresan and Orna-Montesinos, 2021). Academic literacy is an essential element of success for both teachers and students given its role in academic and social life (Cumming, 2013; Englander and Corcoran, 2021; Wu et al., 2020). It provides more job opportunities and the expertise required in academic domains like conference presentations and publications. The term academic literacy refers to one’s ability to understand and control academic discourse conventions of a specific discipline (Wingate, 2012). It generates knowledge and contextualizes learning (Li, 2022). For a long time, academic literacy development was confined to linguistic aspects, but recently a paradigm shift has appeared that situates learning and literacy within the broader context of society, ideology, and power (Cumming, 2013; Li, 2022; Paltridge et al., 2016). This movement assisted educators and researchers to perceive academic literacy development as a social and situated construct that occurs at the intersection of cognitions, emotions, social practices, and macro-societal structures (Canagarajah, 2018; Cumming, 2013). While previous studies on academic literacy development focused on the developmental process of academic literacy of learners (e.g., writing, reading) in different educational contexts (Botha, 2022; Jiang et al., 2010; Kruse, 2013), with the rise of this situated approach to academic literacy, literacy is more considered as the offshoot of several internal (e.g., motivation, attitude, mental health) and external factors (e.g., context, facilities, economy) (Cumming, 2013; Flowerdew, 2013). These complexities demanded a shift toward a more contextualized and embedded approach to academic literacy development (Englander and Corcoran, 2021).

Given its social nature, literacy is now positioned in the context of power relations and macro-societal factors like race, justice, identity, gender, emotion, and access that significantly influence one’s academic literacy development (Canagarajah, 2020). With the spread of English, knowing only local perspectives and norms does not suffice to become academically literate (Bardi, 2015). In many multilingual, multicultural contexts a glocalized perspective beyond local literacy practices is required (Englander and Corcoran, 2019). In such contexts, where many immigrant, multilingual, and international students and teachers try to embed their literacy experiences within larger, social frames, disciplinary skills and requirements from broader geolinguisitic locales are needed (Canagarajah, 2020; Christensen et al., 2005; Leki, 2017). To perform better and deal with different ideological positionings of multilingual and multicultural students, English as a foreign language (EFL) teachers require a contextualized view of academic literacy (Curry et al., 2021). This approach is pivotal for getting familiar with disciplinary and community norms, especially with the emergence and popularity of English for Academic Purposes (EAP) and English Medium Instruction (EMI) in EFL settings (Altbach et al., 2009; Qiu and Fang, 2022; Slaughter and Leslie, 2001).

Although researching academic literacy development is not totally uncharted in L2 contexts, its role and significance in multilingual and multicultural contexts have been limitedly investigated. Moreover, most of the current studies in this domain have focused on the academic literacy development of EFL students, while teachers' perspectives and status have remained under-explored. To fill these gaps, the present theoretical review aimed to present a global view of EFL teachers' academic literacy development, which is, in turn, crucial for students' academic literacy development teachers' academic literacy plays an important role in learners' academic literacy development (Borg, 2019).

## 2 Background

### 2.1 Academic literacy: definitions and approaches

In the pertinent literature, different definitions and explanations exist for the concept of academic literacy. However, the most widely used and comprehensive one is that the term has to do with a gamut of literacy skills associated with content learning and an individual’s higher-order thinking (Shanahan and Shanahan, 2008). It comprises various literacy practices that a learner requires to succeed such as critical and analytical skills, linguistic knowledge, sociocultural awareness, belongingness to an academic community, and language fluency (Kiili et al., 2013). Hence, the concept is not confined to the ability to read and write but entails a range of academic skills. To clarify the construct of academic literacy, different approaches have been offered, to date. Traditional approaches considered academic literacy as a discrete range of linguistic skills that is transferrable to other contexts (Li, 2022). It was more text-driven since language structures were more stressed out during this period (Allison and Harklau, 2010; Fang, 2012). Nevertheless, with the rise of sociocultural theory and socialization theories, the term was positioned and perceived as a social practice that brings about involvement and socialization in academia (Duf, 2010).

“Genre” approach is the next theoretical underpinning on academic literacy, which regards it as a person’s ongoing achievement of common cultural values and communicative practices in a community or field of study (Fisher, 2019). According to this approach, academic literacy can improve academic community functioning given its emphasis on frames of actions (Wenger, 1998). Afterwards, academic literacy shifted from singularity to pluralism with a new approach called “the academic literacies”. This approach is informed by the “genre” approach and complements it. Additionally, the academic literacies approach goes beyond skills and socialization and takes an epistemology and identity stance (Lea and Street, 1998). It fosters access, communication, and identification in academia by echoing social justice and fairness (Gee, 2015). In sum, academic literacy is a dynamic and shifting concept that encompasses various cognitive, linguistic, and sociocultural practices in a given context (Cumming, 2013).

### 2.2 The concept of academic literacy development

Given the significance of academic literacy and literacies in different academic contexts and programs, educators and practitioners paid a special attention to the development of this pivotal construct. This surge of interest led to the emergence of ‘academic literacy development’, which refers to the process of acquiring an understanding of disciplinary practices and requirements and one’s capacity to interpret the relationship between text, context, and social actions of different genres (Bazerman and Prior, 2004). In this sense, academic literacy development concerns knowledge generation, communication, and transformation (Li, 2022). Like academic literacy, the conceptualization of academic literacy development was influenced by different theoretical perspectives. Initially, it was seen as a discrete linguistic skill, then it moved forward to be perceived as a social practice within sociocultural contexts (Cumming, 2013; Gee, 2015).

### 2.3 The dimensions of academic literacy development

**Figure 1:**
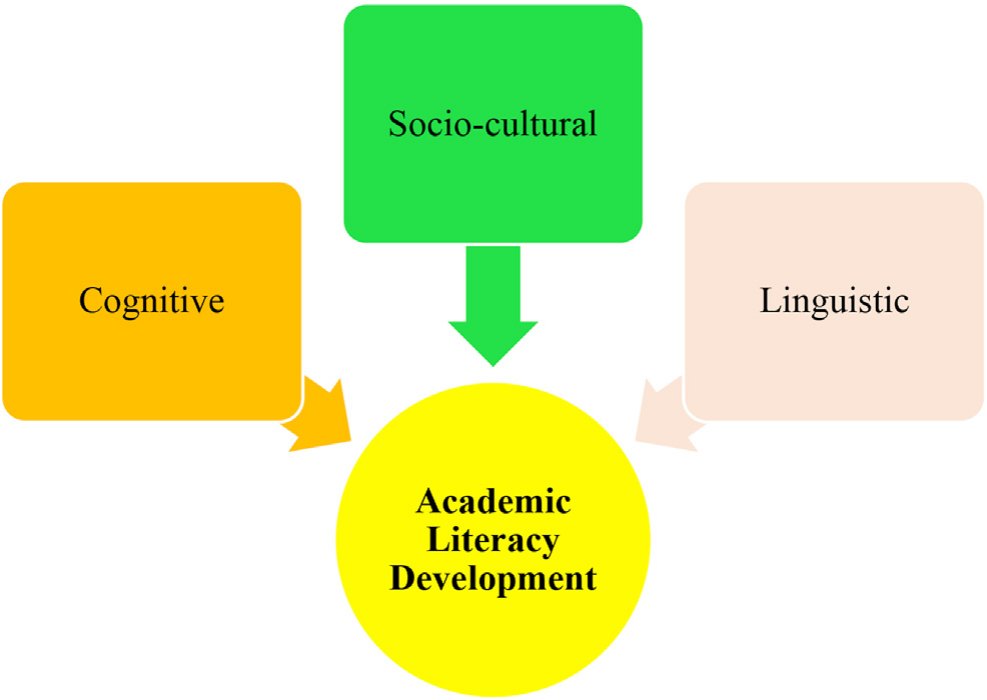
Different dimensions of academic literacy development (Kern, 2000).

After establishing itself in the literature, academic literacy development captured scholarly attention that proved its nature to be multidimensional rather than a single construct (Li, 2022; Short et al., 2011). According to Kern (2000), academic literacy development includes three dimensions; linguistic, cognitive, and socio-cultural (Figure 1). The linguistic dimension regards academic literacy as a linguistic process. It highlights linguistic knowledge, textual organization, genre, and language conventions. The cognitive dimension of academic literacy development considers literacy as an internal and cognitive process that tries to connect knowledge and textual forms in one’s mind. Finally, the socio-cultural dimension places academic literacy in larger societal and cultural conventions and norms. It views academic literacy as a socio-culturally determined practice, which emerges from one’s social interactions (Barton, 2007; Kern, 2000).

These dimensions reveal that the construct of academic literacy development is complex and multi-layered and may demand more research in different contexts to unravel more possible dimensions (Wells, 1987). A probable new dimension, which can be tested scientifically, may be ‘affective dimension’. Many variables and constructs involving learning do approve the role of emotions and affect. However, the trace of ‘affect’ in academic literacy development is yet under-explored. This can be an interesting line of research for further studies. What is clear, to date, is that academic literacy development is multi-dimensional and influenced by context. This is observable in a fresh venue that positions academic literacy development in the context of multilingualism, which has different academic literacy requirements and educational concerns (Christensen et al., 2005).

### 2.4 Academic literacy development in multilingual contexts

With the rise and prominence of the sociocultural approach to academic literacy development, strong premises have been held seeing it as situated and complex. Another emerging feature in this domain is the increasing attention paid to multilingualism and multiculturalism, especially among international students (Li, 2022). This process has been fostered by the globalization era and academic exchange of international students that transformed academic literacy development to a global issue (Caplan and Stevens, 2017). In multilingual contexts, teachers usually encounter several barriers in teaching the English language and the course contents, especially those that demand advanced literacy skills for learning and inquiry (Li, 2018; Okuda and Anderson, 2018; Wang and Derakhshan, 2022). In light of this transition, more attention is required to the development of academic literacy skills of students with various languages and cultures. An upshot of this multilingual vision is the integration of language and cognition into the disciplines in multilingual education (Goldenberg, 2010). Now, the role of teachers has changed to an active one with the agency to make new discoveries and pedagogical practices that reflect their cultural values and languages (Li, 2022). Along with mainstream students, multilingual and multicultural students need to acquire writing, reading, and research skills in a given discipline. Hence, teachers should cultivate in their students' diverse academic literacy skills to project and synthesize information and ideas and communicate effectively. In other words, they need to gain an ‘insider knowledge’ of their own field and discourse community (Soter, 1992). This kind of knowledge is more challenging for non-native English speakers because of its cultural basis (Christensen et al., 2005).

Another area that requires more attention is the research literacy skills that multilingual and multicultural students and teachers need in the globalization era (Derakhshan et al., 2020). In order to find a space for themselves, they need academic literacy skills like having an authorial voice in their publications and cooperating with other community members of their discipline. These are not achievable without an appropriate and professional training course on developing the academic literacies of both teachers and students. Literacy and writing skills are no longer merely linguistic attempts for a better writing, but critical steps to develop an academic voice and identity in multilingual contexts that are characterized by publication pressure (Gee, 2004). All in all, working and studying in multilingual and even bilingual contexts require more academic literacies in the form of a repertoire of biliteracies and multiliteracies that range from writing abilities to awareness of power relations and ideologies in the review processes of academic publication (Palfreyman and Van der Walt, 2017). Depending on disciplines and their requirements, different academic literacies may need to develop in education, which is now internationalized and sticks to global norms.

### 2.5 Previous studies

Academic literacy and its development have been an important aspect of higher education in different fields for decades (Holland, 2019; Wingate, 2018). After establishing a definition and conceptualization, several studies have been done on academic literacy as a separate field that gains its knowledge base from other fields like applied linguistics, anthropology, discourse studies, and sociolinguistics (Lillis and Scott, 2007). This paved the way for others to investigate the features of academic literacy such as one’s capacity to switch practices and genres in line with contextual particularities and control the meanings and identities constructed during these processes (Paltridge and Starfield, 2013). Likewise, the connection between academic literacy and discourse communities has been explored by scholars, who highlighted that academic literacy development depends on one’s ability to accept the culture and identity of the members of the given communities (Ivanič, 1998). Additionally, Wingate (2015) vividly argued that academic literacy development leads to an effective communication in an academic discourse community.

Moreover, a large body of existing studies on academic literacy has focused on different language skills and sub-skills that students can improve in light of receiving literacy training (Fouché et al., 2017; Geisler, 2013; Weideman et al., 2016). With more complementary studies being conducted in this area, it has been proved that academic literacy steps beyond the simple promotion of language skills and many other competencies have stressed. As a case in point, Wilson et al. (2004) contended that academic literacy development has strong interaction and linkage with one’s critical thinking skills. Similarly, Eaton et al. (2017) reiterated the prominence of critical thinking as one of the main features of academic literacy, especially in social sciences.

Some other studies on academic literacy have focused on the impact of specific approaches and practices in improving teachers' and students' academic literacy. For example, Ferenz (2005) investigated the impact of using social media on the development of academic literacy in L2 education. Furthermore, Cheng (2008) examined the role of genre analysis in academic literacy tasks like the analysis of different parts of articles. Moreover, Schwenger (2018) inspected the impact of a new approach to teaching academic literacy called “*Literacy + Numeracy Intervention Process*” on the research abilities of researchers in New Zealand.

Others, however, shifted their attention to teachers' academic literacy as a precondition for learners' academic literacy to occur. For instance, Short and Fitzsimmons (2007) explored teacher-related challenges of developing academic literacy in students and found that the absence of educated teachers and effective teacher training courses are the biggest setbacks. Likewise, Bunch (2013), examined the role of EFL teachers' knowledge and training in their ability to teach literacy skills to students in academia and prepare them for the new educational world. As previous studies indicated, most of the investigations on academic literacy have been limited to the acquisitional process of academic literacy, language skills, and student-related issues in EFL or EAP contexts. In a seminal study in Iran, Zand-Moghadam and Khanlarzadeh (2020) ran a structural equation modeling (SEM) study to disclose the underlying components of academic literacy as perceived by EAP teachers. The results of factor analysis pointed to nine components for the construct of academic literacy, namely (1) familiarity with different genres, (2) familiarity with academic ethics and honesty, (3) familiarity with context and contextual meaning, (4) knowledge of the four language skills, (5) critical thinking ability, (6) familiarity with target discourse community, (7) teachers' familiarity with academic literacy concept and components, (8) scientific article writing ability, and (9) familiarity with computer and technology.

In the context of English for specific purposes (ESP), the construct of teacher academic literacy development has also been sought out by researchers. As a single landmark research, Al-Faraj (2021) carried out a qualitative study on eight ESP teachers in Kuwait using semi-structured interviews, classroom observations, and document collection. The results revealed that ESP teacher perceived academic literacy practices to be context-derived, transferrable and generalizable from one setting to another, communicative, and reflective of social norms of a specific discourse community. Moreover, the ESP teachers contended that their perceptions of academic literacy and academic literacy practices usually shape their pedagogical choices and teaching styles in the classroom. The participants' future visions of the job and professional contexts were regarded as essential for learner engagement in the ESP classroom. Finally, teachers' lack of understanding the situated literacy practices was considered as the main obstacle to teach and develop academic literacies during ESP courses.

In spite of the prominence of all these previously conducted studies, teachers' perceptions and practices of academic literacy development in multilingual and multicultural contexts have mostly remained under-researched, to date. In an influential study, Christensen et al. (2005) examined the efficacy of a model program for multilingual students with academic English problems. The model was called Commanding English (CE), which placed language development within the overall academic content as supported by other faculty members, staff, and advisors, who helped students to build multicultural and multilingual voice and competence. Despite its significance, this line of research is still under-explored. Hence, reviewing the trends in research and practice of academic literacy development is momentous for L2 educators.

### 2.6 Research trends in academic literacy development: past and present trends: a way to the future visions

**Figure 2:**
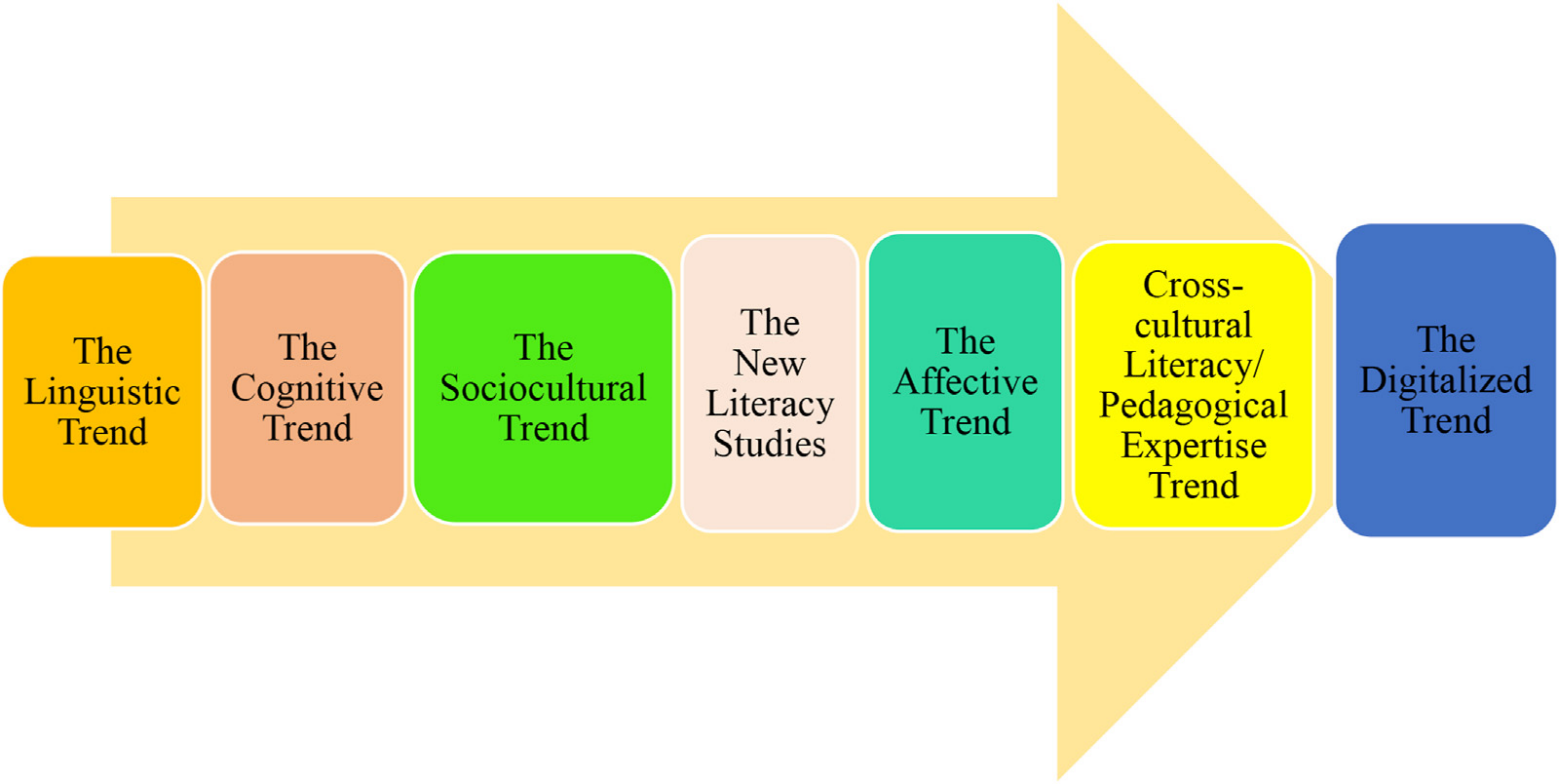
Different trends in researching and practicing academic literacy development.

Research and practice of academic literacy development have gone through different changes and theoretical trends (Figure 2). Traditionally, it was studied and dealt with under the linguistic or language trend that placed emphasis on learning academic skills of reading and writing without errors. This trend is also known as the language-based approach or the text-driven approach that highlighted correct structures and functions in learning the content (Uccelli et al., 2014). It was supported by Systemic Functional Linguistics (SFL), which tried to clarify the links between text and context, linguistic choices, and interpretations in light of specific communicative purposes (Halliday, 2000). Training courses during this trend intended to unveil common structures and communicative functions in the given disciplines (Charles, 2013).

Later, the language-based trend was criticized by cognitivists, who argued that academic literacy development goes beyond a simple acquisition of rhetorical structures and it is a tool for content learning and scientific reasoning (Bailey et al., 2007). This psycholinguistic trend perceived academic literacy development to be connected to one’s cognitive development, knowledge construction, and communication in a discipline (Fang, 2012). It also sought socialization and contextualization of meaning (Granville and Dison, 2005). The cognitive trend accentuated disciplinary-specific ways of knowing, doing, and thinking (Moje, 2015). The problem with this approach to academic literacy development was that it placed a heavy load on the individual’s mind and autonomy in learning discourse competencies in a particular discipline.

Now, the developmental path has come into the sociocultural stance that places academic literacy within larger societal and cultural structures and norms rather than discrete linguistic or cognitive skills (Gee, 2015; Lea and Street, 2006). During this trend, the agency and identity of students and teachers to change community practices are stressed out (Lillis and Scott, 2007). The proponents of this approach consider academic literacy development as a social practice that must echo and care for the individuals' cultural values, beliefs, and ideologies (Moje et al., 2008).

Another trend that grew out of sociocultural perspectives is ‘The New Literacy Studies’ or NLS, which has provided an ideological model of literacy that reconciled between literacy and orality (Chakrabarty, 2020). This trend conceptualized academic literacy as an important social practice that is value-laden, ideological, and influenced by power relations common in social procedures and structures. It was this trend that situated academic literacies models within the power of social discourse to open up innovations and alterations (Lea and Street, 2006).

Although these trends have been insightful enough to move the field forward, future trends may also appear given the development and changes in human knowledge and disciplinary requirement. Previous trends limitedly (if any) consider the role of emotions and affect in the academic literacy development of multilingual and multicultural students, who are experiencing many challenges in learning content and language in L2 contexts. Therefore, a future trend in this domain can be emotion-based as students from international/global contexts require care, love, and devotion on the part of their teachers. Particularly, the role of positive psychology constructs and interpersonal communication factors (love, job devotion, clarity, credibility, stroke, confirmation, etc.) can be inspected in multilingual settings, as well (Wang et al., 2021; Xie and Derakhshan, 2021; Wang et al., 2022). Moreover, the new demands of the globalized world for cross-cultural literacy and pedagogical expertise from teachers can be an emerging trend in this strand of research. In such an era, teachers should use proper techniques in treating and teaching students from diverse education backgrounds. Finally, the utility of technologies and virtual spaces in shaping the academic literacy of students and teachers in multilingual contexts can be unveiled by a digital academic literacy development trend. In today’s digital and digitalized world, teachers and students need new academic literacies in line with technologies. Hence, a digitalized approach to academic literacy development can foster both EFL teachers' pedagogical performance and students' literacies.

## 3 Concluding remarks

In this review article, it was contended that EFL teachers and even students are now requiring different academic literacies in comparison to the decade’s ago. Previously, training courses and research trends on academic literacy and its development were confined to linguistic and cognitive aspects. However, with the globalization of English and the internationalization of higher education, many international students and teachers are now working in multilingual contexts that demand different academic literacies than mainstream educators. In multilingual contexts, teachers may face various challenges in teaching English to students who come from different cultural contexts and speak non-English languages. They need to cultivate multiliteracies in students so that they have an authorial voice in academia and position themselves within their community of practice that adheres to specific academic literacies and rhetorical patterns. Instead of sticking to linguistic aspects of English and the content of education, teachers must focus more on communicative functions and norms that are pivotal in a specific discipline. Since multilingual contexts, especially those in which English is considered as a foreign language, are full of challenges for both teachers and students, educators must strike a balance between language learning and content learning.

By paying attention to these propositions and contentions, EFL teachers can benefit from this theoretical review in that they understand the common trends in teaching English in multilingual and multicultural contexts and propose suitable practices to raise their students' academic literacies. By knowing the requirements of their discipline at the moment, EFL teachers can highlight academic literacies such as academic writing, communicative competence, and digital academic literacies in remote education. They can also improve their mentality about academic literacy development courses in light of the information provided by different trends explained in this article. They may become cognizant that academic literacy development is no longer seen as an isolated construct, but as a social practice sensitive to cultural values and norms.

Moreover, curriculum planners and designers can use this study to modify their current practices and make suggestions to integrate new academic literacies into their curricula at both micro and macro levels in multilingual and multicultural contexts. Teacher trainers in multilingual contexts can use this study as a guide and try to offer professional development courses to L2 teachers with different experiences in which the complexities, challenges, and common academic literacies and norms of their disciplines are highlighted. The trainers must shift from exclusive linguistic aspects of teaching at the multilingual context and capitalize on the social, cultural, emotional, and digital needs of the given discipline. They have to practically instruct the teachers on how to work at multilingual and multicultural milieus so that their pupils can benefit from their knowledge and survive in the globalized world with many academic pressures on non-English speaking students.

Researchers may also find this review insightful in that it extends the literature on academic literacy development in multilingual and multicultural contexts, especially those in which English is spoken and taught as a foreign language. The existing literature on this topic is scan regarding the impact of academic literacy development on L2 education, especially in terms of cultural disparity among students. Furthermore, the current body of knowledge on academic literacy is mostly limited to theoretical studies that intended to explicate what the concept of academic literacy development means, case studies on the development of academic literacies of particular stakeholders in particular educational contexts, one-shot surveys, and quantitative studies on the underlying components of academic literacy development in contexts other that multilingualism and multiculturalism. This is where the present article can be significant and pave the way for future studies that take advantage of other research designs and instruments to present a more vivid picture of academic literacy development in multilingual settings. Additionally, further research can be done on the possible trends like affective trends and digitalized trends to academic literacy development to move the field forward. Needs analysis studies can also be carried out to disclose the literacies that teachers and students of specific fields perceive essential to be covered in training courses. Cross-cultural studies on academic literacies of similar disciplines are also an interesting line of research for future researchers.

## Declarations

### Author contribution statement

All authors listed have significantly contributed to the development and the writing of this article.

### Funding statement

This work was supported by Foundation of Henan Educational Committee [2022-JSJYYB-027].

### Data availability statement

No data was used for the research described in the article.

### Declaration of interest’s statement

The authors declare no conflict of interest.

### Additional information

No additional information is available for this paper.
